# Intrapleural Administration of Hypotonic Cisplatin for Patients With Malignant Pleural Effusions and Non‐Expandable Lungs

**DOI:** 10.1111/1759-7714.70181

**Published:** 2025-11-04

**Authors:** Wataru Mori, Tomoyasu Mimori, Jun Ito, Shun Sorimachi, Shinya Fujioka, Haruki Hirakawa, Yoshihiro Masui, Taichi Miyawaki, Takehito Shukuya, Kazuhisa Takahashi

**Affiliations:** ^1^ Department of Respiratory Medicine Juntendo University Graduate School of Medicine Tokyo Japan

**Keywords:** hypotonic cisplatin treatment, malignant pleural effusion, non‐expandable lungs

## Abstract

**Background/Objectives:**

Thoracostomy and pleurodesis are the mainstay of management for malignant pleural effusions (MPEs). However, pleurodesis may not be effective for patients with MPEs and non‐expandable lungs. Intrapleural chemotherapeutic agents such as hypotonic cisplatin are reportedly useful for treating MPEs with expandable lungs; however, their efficacy in patients with non‐expandable lungs remains unclear. We aimed to analyze the efficacy and safety of intrapleural administration of hypotonic cisplatin in patients with MPEs and non‐expandable lungs.

**Methods:**

We retrospectively analyzed patients with MPEs of thoracic malignancies who were administered intrapleural hypotonic cisplatin. We investigated the changes in drained fluid volume, radiological outcomes at 4 weeks, thoracentesis‐free survival, and adverse events. Between June 2009 and September 2022, 62 patients with MPEs received 69 administrations of hypotonic cisplatin.

**Results:**

The most frequent primary site was the lungs (90.3%), and the mean drained fluid volume per day decreased by 65% (95% confidence interval [CI] 58%–72%) after intrapleural hypotonic cisplatin administration. At 4 weeks post‐administration, MPE volumes decreased in 33 (53.2%) patients, remained unchanged in 22 (35.4%), and increased in seven (11.3%), based on frontal plane chest radiographs. The median thoracentesis‐free survival was 456 days (95% CI, 122–842 days), the 30‐day thoracentesis‐free survival rate was 86.1%, and the 90‐day survival rate was 70.8%. In total, 37 patients (59.7%) were censored. The most frequent adverse event was pleural empyema, observed in four patients.

**Conclusions:**

Intrapleural hypotonic cisplatin administration decreased or stabilized pleural effusion and may be useful for suppressing MPE with non‐expandable lungs.

ClinicalTrials.gov identifier: E23‐0003.

## Graphical abstract

Intrapleural administration of hypotonic cisplatin (HPT) decreased or stabilized pleural effusions and was well tolerated in patients with malignant pleural effusion and non‐expandable lungs.

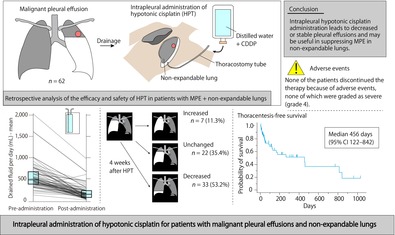

## 1 Introduction

Malignant pleural effusion (MPE) is a common complication of advanced‐stage malignancies and typically indicates poor prognosis [1, 2]. Lung cancer is the leading cause of MPE, accounting for 37.5% of all cases [3]. The primary goals of MPE management are to alleviate symptoms and improve the quality of life rather than to prolong survival [4]. For most symptomatic patients, therapeutic thoracentesis, with or without thoracostomy, is initially performed, followed by chemical pleurodesis using inflammatory agents, such as talc, antibiotics, antiseptics, and microorganisms, to achieve long‐term control of MPE [5, 6].

Pleural thickening, endobronchial obstruction, and chronic atelectasis can hinder complete lung expansion, resulting in a non‐expandable lung [7]. In such cases, the visceral and parietal pleurae fail to appose even following drainage of the pleural effusion, rendering patients with non‐expandable lungs unsuitable candidates for chemical pleurodesis [8]. This condition is known to complicate more than 50% of all MPE cases [9], limiting treatment options for this patient subgroup. The mainstay of management remains repeat thoracentesis, which provides only temporary symptom relief or the use of an indwelling pleural catheter (IPC) [5]. Other interventions, such as pleuroperitoneal shunting and pleurectomy, are invasive and rarely performed [10, 11]. Therefore, finding more definitive and less invasive treatment options for patients with non‐expandable lungs remains a significant challenge.

Ichinose et al. reported that intraoperative intrapleural hypotonic cisplatin suppressed pleural dissemination and effusion in patients with non‐small cell lung cancer [12]. In a prospective study, Seto et al. reported that intrapleural administration of hypotonic cisplatin via thoracostomy tube, or hypotonic cisplatin treatment (HPT) thereafter, effectively controlled MPE in patients with expandable lungs [13]. However, little is known about the efficacy of this treatment in patients with MPEs and non‐expandable lungs.

This study aimed to retrospectively analyze the efficacy and safety of intrapleural administration of hypotonic cisplatin in patients with MPE, most of whom had complications due to non‐expandable lungs.

## 2 Methods

### 2.1 Patients and Clinical Data

**FIGURE 1:**
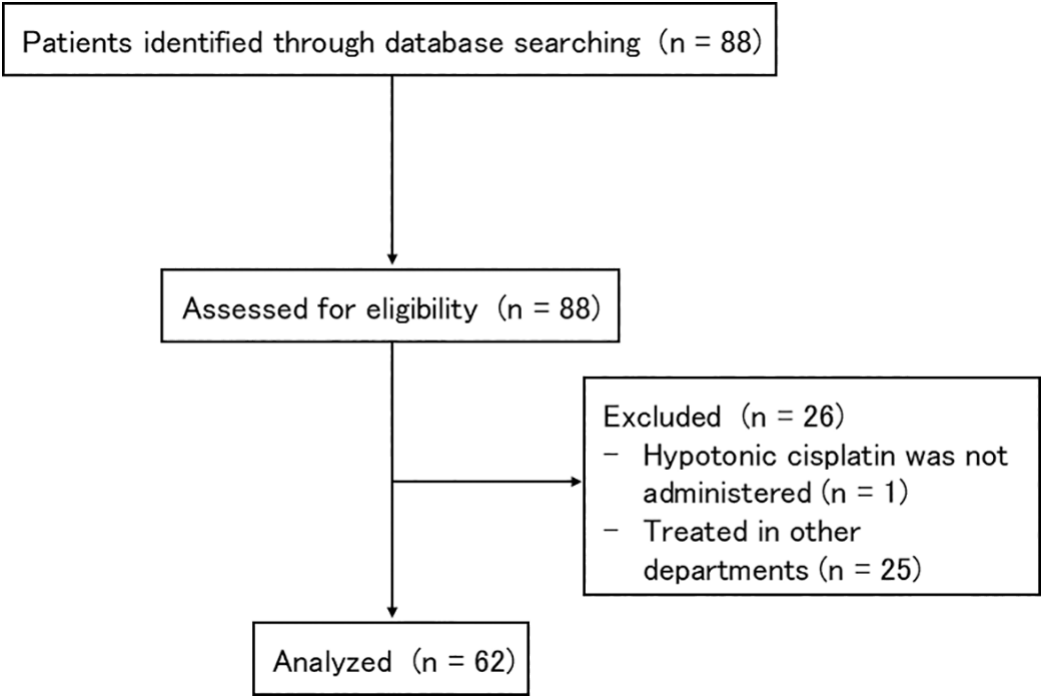
Flowchart of patient selection.

We conducted a retrospective review of consecutive patients with thoracic malignancies complicated by MPEs who were treated with HPT between April 2009 and September 2022 at the Department of Respiratory Medicine, Juntendo University Hospital, Tokyo, Japan. A flowchart of the patient selection is shown in Figure 1. This study investigated the efficacy and safety of HPT. MPE was diagnosed based on positive cytological examination of the pleural effusion [14]. Patients who were unable to achieve full lung expansion post‐thoracostomy were classified as having non‐expandable lungs. Baseline patient characteristics, including the primary cancer site, history of systemic chemotherapy, creatinine clearance (CrCl), estimated using the Cockcroft and Gault formula, drained fluid volume per day, and adverse events following the procedure, were collected from the patients' medical records. Off‐label use of cisplatin was approved by the Ethics Committee of Juntendo University Hospital. This study was registered with the University Hospital Medical Information Network Clinical Trials Registry (E23‐0003).

### 2.2 Evaluating the Response to HPT

To evaluate the response to HPT, we assessed the changes in the volume of drained fluid per day, serial frontal plane chest radiographs, and thoracentesis‐free survival. The mean volumes of drained fluid pre‐ and post‐HPT were compared. Thoracentesis‐free survival was defined as the duration from hypotonic cisplatin administration to death or the need for thoracentesis due to symptomatic recurrence of MPE. Adverse events were graded according to the Common Terminology Criteria for Adverse Events version 5.0 [15]. In addition, given the nephrotoxic nature of cisplatin [16], we evaluated the association between baseline renal function and adverse events.

### 2.3 Definition of the Lung Expansion Scale

To quantify the degree of lung expansion on frontal plane chest radiographs, the Lung Expansion Scale (LES) was defined as follows. The affected side of the lung field was divided into three sections using horizontal lines. Each section was scored as follows: 1 point if the lung was completely collapsed; 0.5 points if the relevant lung portion partially collapsed; and 0 points, if it fully expanded. We calculated the total score of the three sections to provide a scale ranging from 0 (no pleural effusion) to 3 (white‐out). Figure S1 illustrates the calculation of LES. Two independent researchers (WM and TM) evaluated the plane chest radiographs. A lung was classified as expandable if the LES score was 0 and as non‐expandable if the LES score was > 0.

### 2.4 Thoracostomy and Intrapleural Administration of Hypotonic Cisplatin

Thoracostomy was performed using a double‐lumen chest tube and sufficient pleural effusion was removed. After premedication with 1% lidocaine, 25 mg of cisplatin was infused into 500 mL of distilled water. The cisplatin dosage was based on the dose confirmed to be safe in a prospective study involving patients with MPE and expandable lungs [13]. The patients were instructed to change their positions intermittently for 1 h when the thoracostomy tube was clamped. The drainage tube was removed if the volume of the drained fluid decreased to ≤ 200 mL/day.

### 2.5 Statistical Analyses

Statistical analyses were performed using JMP version 18.0. Differences in drained fluid volumes and LES were assessed using the Wilcoxon signed‐rank test. The median thoracentesis‐free survival and corresponding 95% confidence intervals (CIs) were estimated using the Kaplan–Meier method. Hazard ratios and the corresponding 95% CIs were estimated using the Cox proportional‐hazards models, and the log‐rank test was used to compare between the groups. The independence of adverse event frequency and CrCl level was evaluated using the chi‐square test. A *p*‐value < 0.05 was considered statistically significant.

## 3 Results

### 3.1 Patient Characteristics

During the study period, 62 patients with MPE underwent 69 intrapleural administrations of hypotonic cisplatin. The baseline patient characteristics are summarized in Table 1. The median patient age was 70.5 years (range, 41–89 years), and 43 (69.3%) patients were men. Lung cancer was the most common primary diagnosis, accounting for 90.3% of cases, followed by malignant pleural mesothelioma (4.8%), cancer of unknown primary origin (4.8%), and thymic carcinoma (3.2%). The right side of the pleural spaces was affected in 41 (66.1%) patients. Talc was used concomitantly in eight (12.9%) patients. In addition, 55 (88.7%) patients had previously received systemic chemotherapy and 29 (46.8%) had received chemotherapy after undergoing HPT.

**TABLE 1:** Patient characteristics.

	*n* (%)
Sex	
Female	19 (30.7)
Male	43 (69.3)
Age (years)	
Range	41–89
Median	70.5
Pathological diagnosis	
Non‐small cell lung cancer	55 (88.7)
Small‐cell lung carcinoma	1 (1.6)
Malignant pleural mesothelioma	3 (4.8)
Thymic carcinoma	2 (3.2)
Unknown origin	3 (4.8)
Creatinine clearance (mL/min)	
Range	19.6–184.3
Median	85.9
< 60 mL/min	16 (25.8)
Laterality	
Left	21 (33.9)
Right	42 (66.1)
Concomitant use of talc	8 (12.9)
History of systemic chemotherapy	
Overall	55 (88.7)
Post‐intrapleural administration of hypotonic cisplatin	29 (46.8)

### 3.2 Volume of Drained Fluid Per Day

**FIGURE 2:**
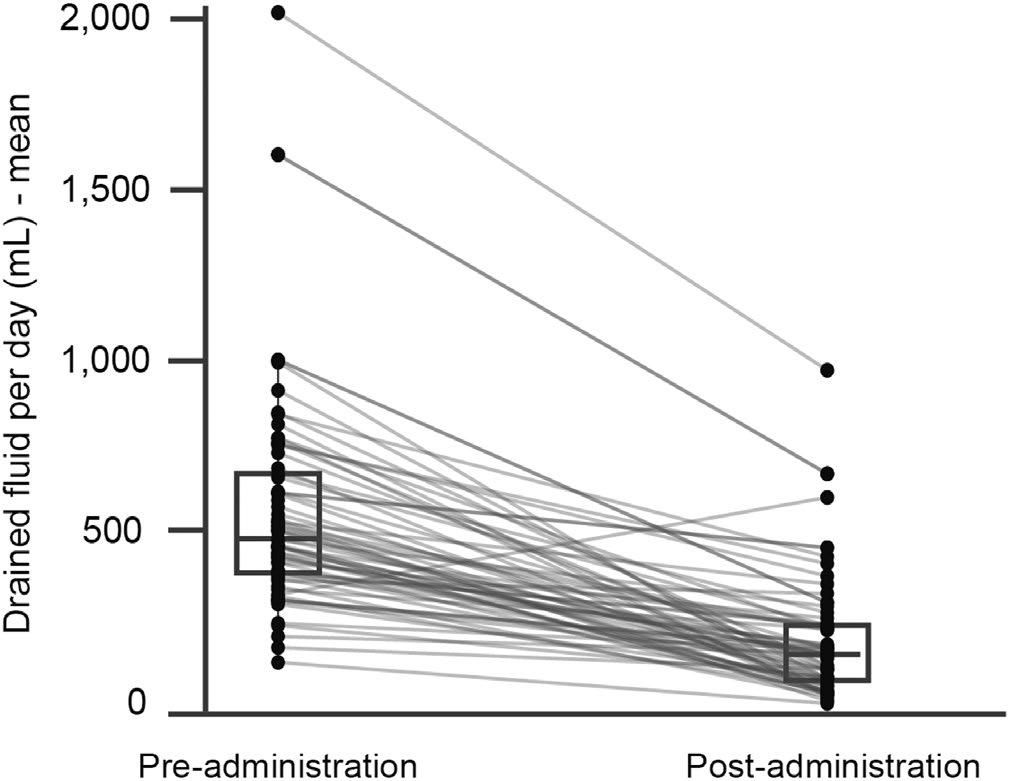
Changes in the mean volume of drained fluid. Parallel plot of the mean volume of drained fluid. Each pair of dots linked in a line corresponds to a specific patient.

The changes in the mean volume of the drained fluid are shown in Figure 2. The median duration of chest tube placement was 10 days (range, 5–37). HPT was performed at a median of 6 days (range, 1–20) following the initial intervention. Post‐HPT, the mean volume of drained fluid per day significantly decreased by 383.5 mL (95% confidence interval [CI] 322.1–445.0 mL, *p* < 0.0001), which was equivalent to a reduction of 65% (95% CI 58%–72%, *p* < 0.001).

### 3.3 Radiographic Outcome

Changes in the LES scores are summarized in Table 2.

**TABLE 2:** Distributions of the LES scores.

Score	Pre‐thoracostomy *n* (%)	Pre‐infusion *n* (%)	Four weeks post‐infusion
0	0 (0)	4 (6.7)	0 (0)
0.5–1.5	22 (35.4)	57 (91.7)	35 (56.5)
2–3	40 (64.6)	1 (1.6)	27 (43.5)

Abbreviation: LES, lung expansion scale.

**FIGURE 3:**
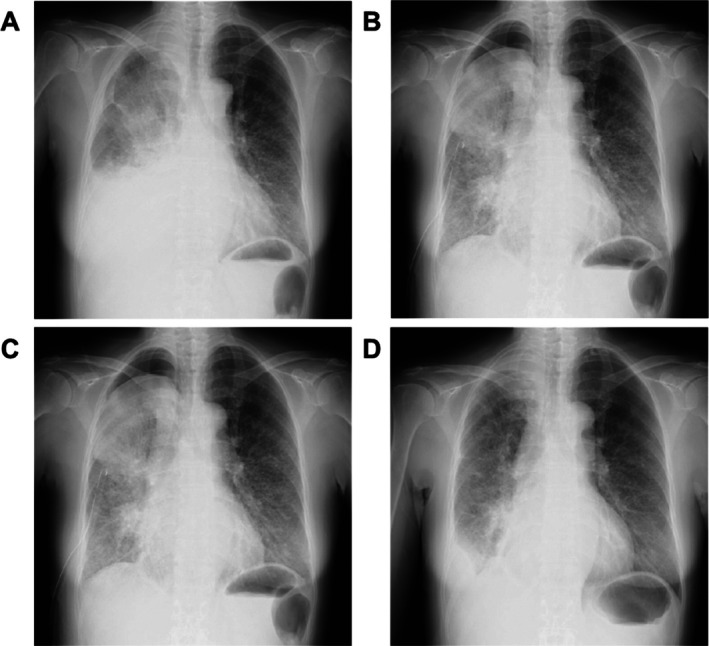
A representative case of serial frontal plane chest radiographs. This series depicts the lungs of a 50‐year‐old woman with lung adenocarcinoma. (A) Before tube thoracostomy; (B) immediately before intrapleural hypotonic cisplatin administration; (C) at 4 weeks; (D) at 8 weeks post‐administration. The LES score was two points pre‐thoracostomy, subsequently decreased to 1.5 just before the intrapleural administration of hypotonic cisplatin, and further decreased to 1 at 4 weeks post‐administration. LES, lung expansion scale.

At baseline, the mean LES score was 2.1 Before hypotonic cisplatin administration, only four (6.5%) patients achieved an LES of 0 or full expansion of the lung; however, 58 (93.5%) patients had an LES score of > 0, indicating a non‐expandable lung. At 4 weeks post‐HPT, the LES score decreased in 33 (53.2%) patients, remained unchanged in 22 (35.4%), and increased in seven (11.3%). The mean LES score reduction at 4 weeks post‐HPT was 0.46 (95% CI −0.66–−0.26, *p* < 0.0001). The reduction in LES score was not significantly different between patients with and without any history of systemic chemotherapy (*p* = 0.22). A representative example is shown in Figure 3.

### 3.4 Thoracentesis‐Free Survival

**FIGURE 4:**
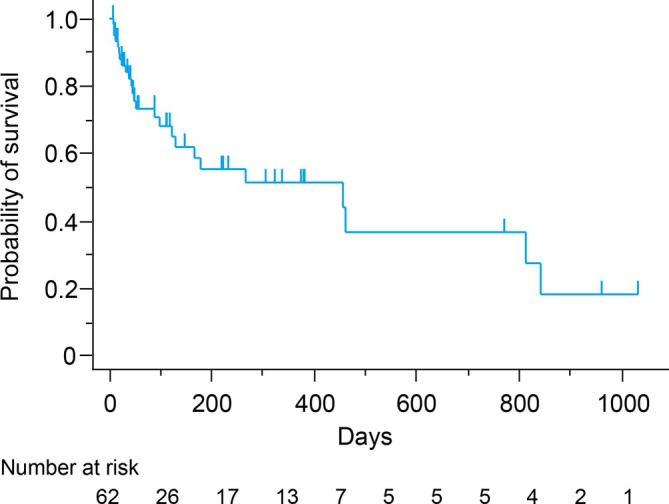
Thoracentesis‐free survival. Kaplan–Meier estimates of thoracentesis‐free survival for all patients.

The median thoracentesis‐free survival time was 456 days (95% CI, 122–842 days) (Figure 4). The estimated 30‐day thoracentesis‐free survival rate was 86.1% and the 90‐day survival rate was 70.8%. In total, 37 (59.7%) patients were lost to follow‐up and were censored (Figure S2). All censored patients were referred to chronic or palliative care settings. History of systemic chemotherapy after the administration of hypotonic cisplatin was associated with better thoracentesis‐free survival (hazard ratio, 0.15; 95% CI 0.05–0.40, *p* < 0.001) (Figure S3).

### 3.5 Adverse Events

The adverse events and their grades are shown in Table 3. The most common adverse event was pleural empyema, which was observed in four (6.5%) patients. Moreover, interstitial pneumonia, skin metastases, infection at the chest tube drainage site, acute exacerbation of interstitial pneumonia, fever, and nausea/vomiting were observed in descending order of frequency. Ototoxicity and nephrotoxicity were not observed in this study. There was no significant difference in the overall frequency of adverse events between patients with CrCl < 60 mL/min (31.3%) and those with CrCl ≥ 60 mL/min (23.9%).

**TABLE 3:** Adverse events.

Grade	1	2	3	4	Total (*n*, %)
Empyema	0	0	4	0	4 (6.5)
Interstitial lung disease	1	2	0	0	3 (4.8)
Drainage site infection	0	2	0	0	2 (3.2)
Skin metastasis	2	0	0	0	2 (3.2)
Exacerbation of interstitial pneumonia	0	0	1	0	1 (1.6)
Pneumonia	0	0	1	0	1 (1.6)
Nausea/vomiting	0	1	0	0	1 (1.6)
Hemothorax	0	0	1	0	1 (1.6)

## 4 Discussion

To the best of our knowledge, this study is the first to report improved control of MPEs in patients with non‐expandable lungs following intrapleural administration of hypotonic cisplatin. In this study, administration of hypotonic cisplatin resulted in either decreased or stable pleural effusion volumes in patients with MPE, most of whom had non‐expandable lungs. The volume of the drained fluid decreased by 65%, and plane chest radiographs indicated decreased or stable pleural effusion volumes at 4 weeks post‐intervention. The median thoracentesis‐free survival was 456 days, with a 90‐day thoracentesis‐free survival rate of 70.8%. HPT was generally well tolerated, with no patient discontinuing therapy because of adverse events, none of which were graded as severe (grade 4).

We hypothesized that two primary mechanisms underlie the efficacy of intrapleural hypotonic cisplatin. First, hypotonicity may exert cytotoxic effects and enhance the activity of chemotherapeutic agents. In vitro experiments have demonstrated that distilled water combined with cisplatin decreases the viability of tumor cells compared to saline solution coupled with cisplatin [12]. Second, the chemotherapeutic agents themselves may have a direct cytotoxic effect on tumor cells. Animal experiments have shown that intraperitoneal cisplatin or carboplatin increases the tumor platinum concentration and tumor growth delay [17, 18]. Local administration of chemotherapeutic agents can confer a survival benefit in abdominal malignancies, particularly epithelial ovarian cancer (EOC) [19, 20] and possibly gastric cancer [21]. It is plausible that intrapleural chemotherapy has similar functions. However, to date, no studies have demonstrated a survival benefit, specifically for intrapleural chemotherapy [5]. This may be partly attributable to the fact that EOC is generally confined to the abdominal cavity, making it an ideal target for intraperitoneal chemotherapy [22]. However, the efficacy of intrapleural chemotherapy remains unclear.

Intrapleural hypotonic cisplatin administration may have multiple roles in managing MPEs in non‐expandable lungs and improving the quality of life by providing symptomatic relief and reducing the need for thoracentesis [23]. In this study, the MPE volumes were stable or decreased in the majority of patients at 4 weeks after HPT. The reduction in LES was not significantly different between patients with and without a history of systemic chemotherapy. These findings suggest that HPT may be effective irrespective of the administration of systemic chemotherapy at least in the short term following HPT. This was in contrast to the thoracentesis‐free survival, which was significantly superior in patients who received post‐HPT chemotherapy. A reduction in the drained fluid volume could facilitate clinicians in removing the tube. Furthermore, it may facilitate pleurodesis by suppressing pleural effusion production, thereby helping to dry the pleural space prior to the application of a sclerosant, which is necessary for successful pleurodesis. The short‐term effects of HPT have the potential to enhance the quality of life of patients diagnosed with MPE and non‐expandable lungs.

This study has certain limitations. Due to its retrospective nature, small sample size, and single‐arm design, a causal relationship between intrapleural hypotonic cisplatin use and improved MPE control could not be established. This study was conducted at a single institution, limiting its generalizability and applicability. Moreover, more than half of the patients were censored, which may have introduced bias into the survival analysis. In Japan, a significant number of patients with advanced‐stage cancer experience a transition from acute care hospitals to chronic care settings. This transition was the primary reason for the termination of follow‐up in our patients. The lack of laboratory data on post‐administration plasma cisplatin concentrations implies that it remains unclear whether HPT is safe, regardless of baseline renal function. Finally, the effect of systemic chemotherapy was not sufficiently addressed, which could have biased the true efficacy of HPT. It is necessary to have a proper control arm to balance confounding factors, including the history of chemotherapy. A prospective, large‐scale study with a parallel control group is necessary to address these limitations. Although chemical pleurodesis using talc is the preferred intervention for patients with MPE and expandable lungs [23], its efficacy in patients with non‐expandable lungs has not been fully examined. Therefore, clinical trials assessing the effectiveness of talc pleurodesis with and without HPT are needed. Nevertheless, this study, which utilized real‐world data from consecutive patients, demonstrates the potential value of HPT and provides a basis for further studies.

Currently, the treatment options for MPE with non‐expandable lungs are limited to repeat thoracentesis or IPC, and symptom control remains suboptimal. We believe that intrapleural hypotonic cisplatin has the potential to play a significant role in the management of MPE.

## 5 Conclusion

Intrapleural administration of hypotonic cisplatin leads to decreased or stable pleural effusions and may be useful for suppressing MPE with non‐expandable lungs.

## Author Contributions

Conceptualization: Wataru Mori, Tomoyasu Mimori, Jun Ito, and Takehito Shukuya. Methodology: Wataru Mori, Tomoyasu Mimori, and Jun Ito. Validation: Wataru Mori and Tomoyasu Mimori. Formal analysis: Wataru Mori and Tomoyasu Mimori. Investigation: Wataru Mori and Tomoyasu Mimori. Data curation: Wataru Mori, Tomoyasu Mimori, Shun Sorimachi, Shinya Fujioka, Haruki Hirakawa, and Yoshihiro Masui. Writing – original draft preparation: Wataru Mori. Writing – review and editing: Tomoyasu Mimori, Jun Ito, Shinya Fujioka, Haruki Hirakawa, Yoshihiro Masui, Taichi Miyawaki, Takehito Shukuya, and Kazuhisa Takahashi. Supervision: Tomoyasu Mimori. Project administration: Tomoyasu Mimori, Taichi Miyawaki, and Kazuhisa Takahashi. All authors have read and agreed to the published version of the manuscript.

## Ethics Statement

The study protocol was approved by the Ethics Committee of the institution and was conducted according to the Declaration of Helsinki.

## Consent

The Ethics Committee of each participating hospital waived the requirement for the investigator to obtain a signed consent form from the participants.

## Conflicts of Interest

The authors declare no conflicts of interest.

## Supporting information


**Figure S1:** Illustrative examples of the LES score.
A. LES of 0 (no effusion).
B. LES score of 1 (upper‐third, 0; middle‐third, 0.5; lower‐third, 0.5).
C. LES score of 2 (upper‐third, 0; middle‐third, 1; lower‐third, 1).
D. LES score of 3 (white‐out).
Abbreviation: LES, Lung Expansion Scale.

**Figure S2:** Event plot.
The time to failure or censoring is presented for each patient. The solid line represents the period during which the patient was confirmed to be functioning, and the dashed line represents the period during which the functional status was unknown. Patients who received tyrosine kinase inhibitors are indicated by green lines.

**Figure S3:** Thoracentesis‐free survival for patients with or without a history of post‐infusion chemotherapy.
Kaplan–Meier estimates of thoracentesis‐free survival in patients with or without a history of systemic chemotherapy after intrapleural administration of hypotonic cisplatin.

## Data Availability

The data that support the findings of this study are available on request from the corresponding author. The data are not publicly available due to privacy or ethical restrictions.
