# Supplementary figures and images for: Intrapleural Administration of Hypotonic Cisplatin for Patients With Malignant Pleural Effusions and Non‐Expandable Lungs
Source: Thorac Cancer. 2025 Nov 4;16(21):e70181. doi: 10.1111/1759-7714.70181 (PMC12585921; doi:10.1111/1759-7714.70181)

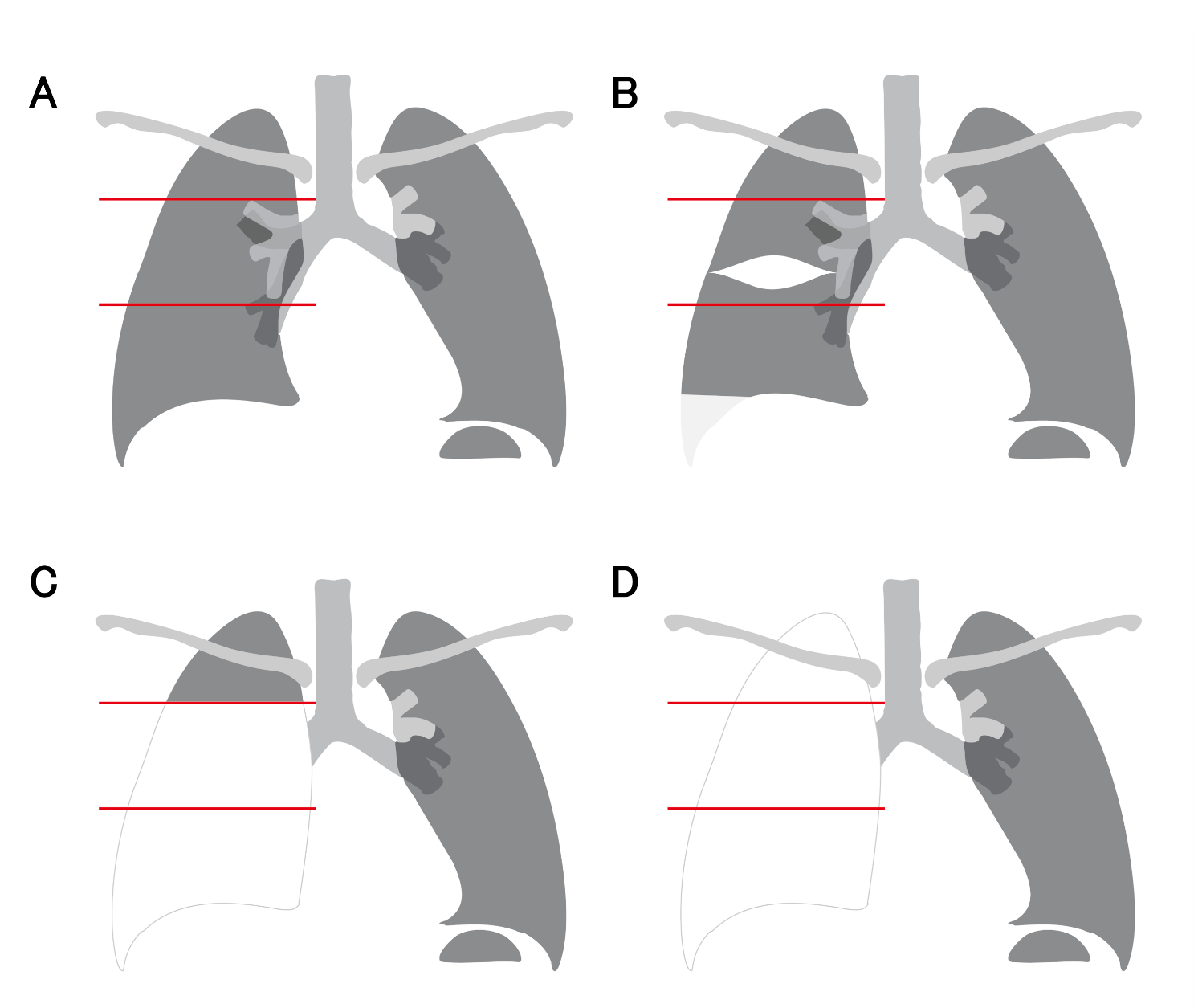

Supplement: Supplementary file 1 — Figure S1: Illustrative examples of the LES score. A. LES of 0 (no effusion). B. LES score of 1 (upper‐third, 0; middle‐third, 0.5; lower‐third, 0.5). C. LES score of 2 (upper‐third, 0; middle‐third, 1; lower‐third, 1). D. LES score of 3 (white‐out). Abbreviation: LES, Lung Expansion Scale. [file TCA-16-e70181-s001.docx]

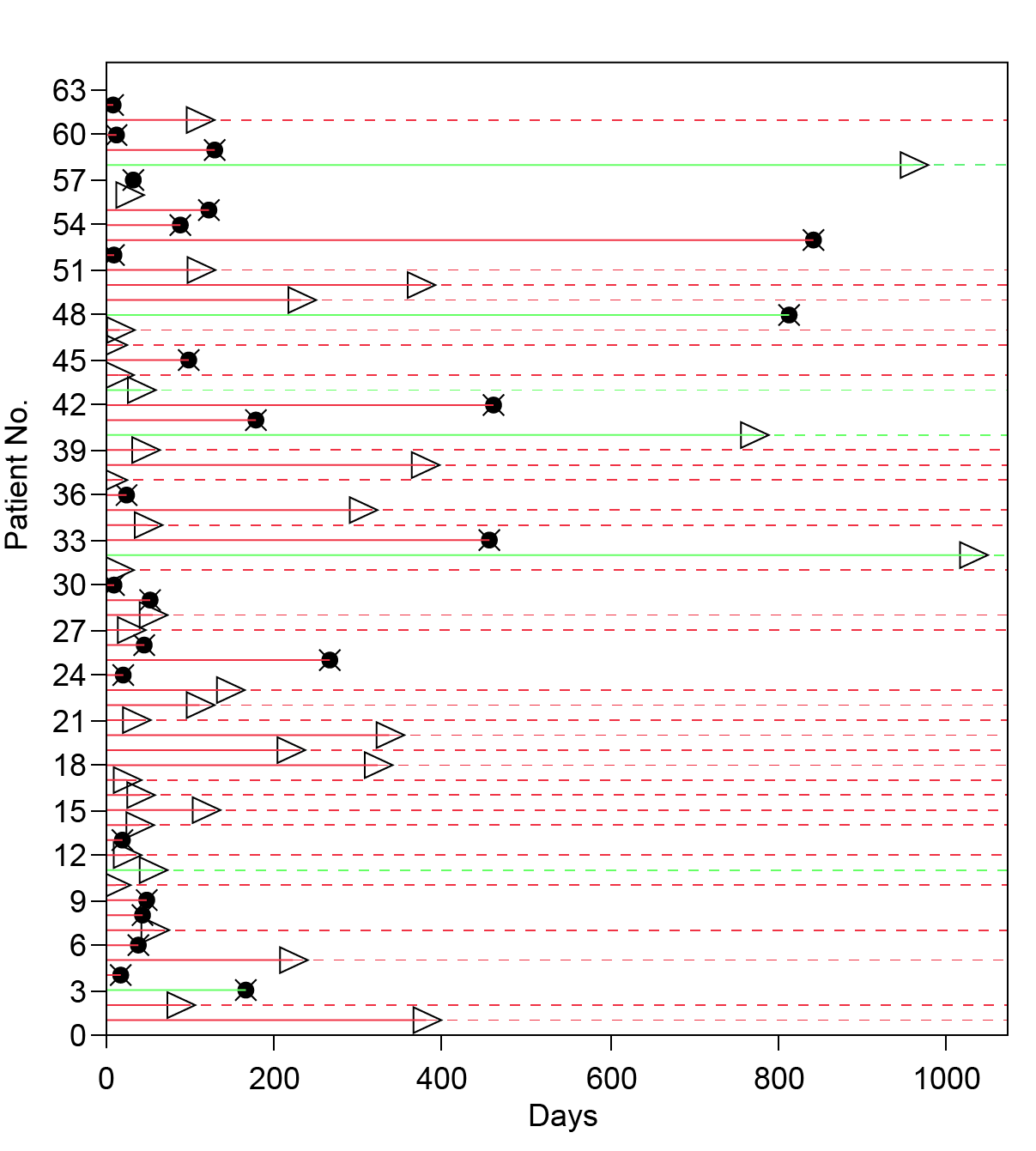

Supplement: Supplementary file 2 — Figure S2: Event plot. The time to failure or censoring is presented for each patient. The solid line represents the period during which the patient was confirmed to be functioning, and the dashed line represents the period during which the functional status was unknown. Patients who received tyrosine kinase inhibitors are indicated by green lines. [file TCA-16-e70181-s003.docx]

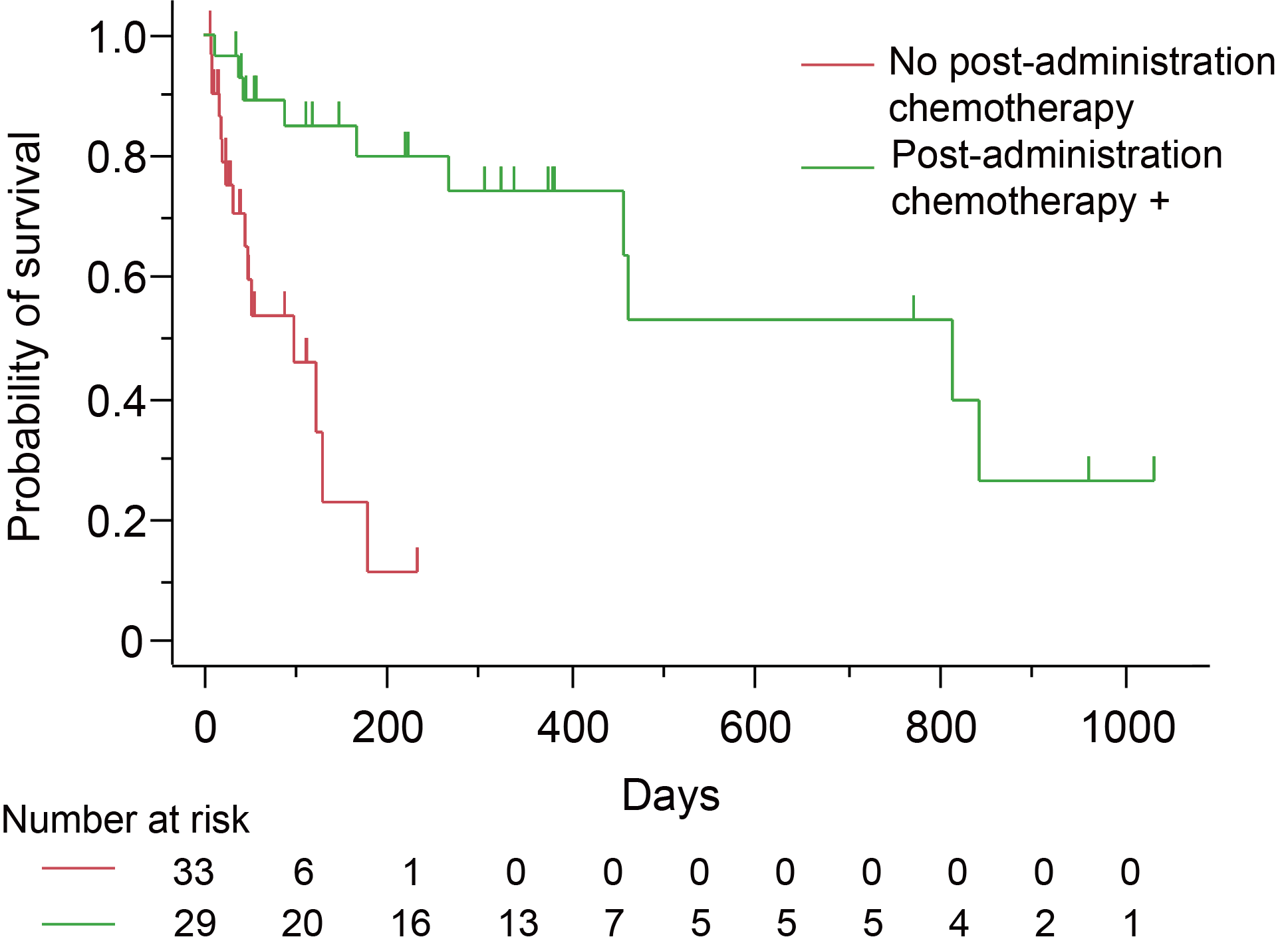

Supplement: Supplementary file 3 — Figure S3: Thoracentesis‐free survival for patients with or without a history of post‐infusion chemotherapy. Kaplan–Meier estimates of thoracentesis‐free survival in patients with or without a history of systemic chemotherapy after intrapleural administration of hypotonic cisplatin. [file TCA-16-e70181-s002.docx]
